# Orally Administered Lactobacilli Strains Modulate Alveolar Macrophages and Improve Protection Against Respiratory Superinfection

**DOI:** 10.3390/biom14121600

**Published:** 2024-12-14

**Authors:** Leonardo Albarracin, Stefania Dentice Maidana, Kohtaro Fukuyama, Mariano Elean, Julio Nicolás Argañaraz Aybar, Yoshihito Suda, Keita Nishiyama, Haruki Kitazawa, Julio Villena

**Affiliations:** 1 Laboratory of Immunobiotechnology, Reference Centre for Lactobacilli (CERELA-CONICET), San Miguel de Tucumán 4000, Argentina; lalbarracin@herrera.unt.edu.ar (L.A.); stefi.dentice@gmail.com (S.D.M.); melean@cerela.org.ar (M.E.); 2 Food and Feed Immunology Group, Laboratory of Animal Food Function, Graduate School of Agricultural Science, Tohoku University, Sendai 980-8572, Japan; kotaro.fukuyama.p8@dc.tohoku.ac.jp (K.F.); keita.nishiyama.a6@tohoku.ac.jp (K.N.); 3 Cátedra de Inmunología, Instituto de Microbiología, Facultad de Bioquímica, Química y Farmacia, Universidad Nacional de Tucumán, San Miguel de Tucuman 4000, Argentina; nic0laz@hotmail.com; 4 Department of Food, Agriculture and Environment, Miyagi University, Sendai 980-8572, Japan; suda@myu.ac.jp; 5 Livestock Immunology Unit, International Education and Research Centre for Food and Agricultural Immunology (CFAI), Graduate School of Agricultural Science, Tohoku University, Sendai 980-8572, Japan

**Keywords:** respiratory immunity, superinfection, probiotic lactobacilli, alveolar macrophages, *Lacticaseibacillus rhamnosus* CRL1505

## Abstract

Orally administered immunomodulatory lactobacilli can stimulate respiratory immunity and enhance the resistance to primary infections with bacterial and viral pathogens. However, the potential beneficial effects of immunomodulatory lactobacilli against respiratory superinfection have not been evaluated. In this work, we showed that the feeding of infant mice with *Lacticaseibacillus rhamnosus* CRL1505 or *Lactiplantibacillus plantarum* MPL16 strains can reduce susceptibility to the secondary pneumococcal infection produced after the activation of TLR3 in the respiratory tract or after infection with RVS. The treatment of mice with CRL1505 or MPL16 strains by the oral route improved the production of interferons in the respiratory tract, differentially modulated the balance of pro- and anti-inflammatory cytokines, reduced bacterial replication, and diminished lung damage. Additionally, we demonstrated that orally administered lactobacilli confer longstanding protection against secondary *Streptococcus pneumoniae* infection and that this effect would be mediated by the stimulation of trained alveolar macrophages. This work contributes to revealing the mechanisms involved in the modulation of the gut–lung axis by beneficial microbes by demonstrating that specific lactobacilli strains, through the stimulation of the common mucosal immune system, would be able to support the development of trained alveolar macrophages that would confer longstanding protection against secondary bacterial challenges produced after a primary inflammatory event in the respiratory mucosa.

## 1. Introduction

Respiratory infections are among the most frequent diseases in humans. Despite the advances in biomedical research and the development of antibiotics, vaccines, and medical support, the hospitalizations and mortality associated with respiratory infections are still high in the global population [1]. Notably, respiratory superinfections that are produced by a secondary bacterial challenge after a primary virus infection are the most important cause of excessive mortality and hospitalization [1,2]. Pathogens frequently associated with respiratory superinfections are influenza virus (IFV) and respiratory syncytial virus (RSV), while *Streptococcus pneumoniae* is a bacterium commonly identified as an agent of secondary bacterial pneumonia [3,4]. Studies have revealed that the immune system of the host has an important role in the outcome of respiratory infections, both in positive and negative means. While the respiratory mucosa’s immune cells are crucial in eliminating pathogens and providing long-lasting immunity, they may also conduct to detrimental inflammation, generating lung tissue alterations [5]. The appropriate regulation of the immune system becomes particularly necessary in the context of respiratory superinfections, since the immunopathology induced by viral infections conditions the immune response against secondary bacterial pneumonia. Thus, strategies that help to modulate the immune responses against primary viral infections as well as secondary bacterial challenges are actively searched for in order to diminish the incidence and severity of respiratory superinfections.

Research has shown that the gut microbiota has a key role in maintaining immunity in the respiratory mucosa by regulating responses both under normal conditions and in front of infections. Notably, the gut microbiota significantly influences respiratory innate antiviral defenses through its capability to regulate the function of respiratory epithelial cells, dendritic cells, and macrophages (reviewed in [6]). Furthermore, research over the last decade has conclusively demonstrated that it is possible to use specific strains of beneficial microbes, orally administered, to regulate respiratory immunity and increase resistance to infections [7]. Among various lactobacilli strains evaluated by our laboratory considering their ability to modulate the mucosal immune system, we determined that orally administered *Lacticaseibacillus rhamnosus* CRL1505 has a remarkable capacity to enhance respiratory immunity [6]. Adult immunocompetent mice fed the CRL1505 strains exhibited significantly higher resistance to the respiratory infection with the Gram-positive pathogen *Streptococcus pneumoniae* [6]. We also performed studies in infant mice models with two respiratory viruses: the respiratory syncytial virus (RSV) and influenza virus (IFV) [6,8]. We observed that the feeding of mice with the CRL1505 strain significantly diminished lung viral replication, reduced tissue damage, and improved survival in response to RSV or IFV [6] challenges. The improved protection against viral infections induced by *L. rhamnosus* CRL1505 were related to a differential expression of interferons (IFNs) and antiviral factors and to a distinct balance of pro- and anti-inflammatory factors in the respiratory tract. Furthermore, we conducted studies to assess the impact of *L. rhamnosus* CRL1505 on the health of children under five years old. We studied how a probiotic yogurt containing the CRL1505 strain influenced children’s immunity and examined its effects on respiratory infections in terms of frequency and severity [9]. The results showed a significant decline in the incidence and severity of respiratory infections in children who received the probiotic treatment compared to the placebo group.

We also aimed to characterize the immunological mechanisms implicated in the effect of the CRL1505 strain and observed that this probiotic bacterium can modulate the common mucosal immune system. The small intestine’s immune system is connected to the systemic and other mucosal immune systems through lymphatic and blood circulation. Therefore, an immune response initiated in the gut can disseminate systemically, reaching other mucosal and non-mucosal sites, including the respiratory tract [10]. Our research has shown that mobilization of CD4^+^ T cells from the gut to the lung and increases in IFN-γ in the respiratory mucosa play crucial roles in *L. rhamnosus* CRL1505′s capacity to modulate AMph (AMph) activity, which in turn enhances the response to bacteria and virus infection. In our experiments, the use of anti-IFN-γ or anti-CD4 antibodies or the depletion of AMphs completely abolished the capacity of the *L. rhamnosus* CRL1505 to modulate respiratory immunity and protect against pathogens [11].

In the past years, we have made efforts, on the one hand, to understand the cellular and molecular interactions between the CRL1505 strain and the host that are involved in the regulation of immunity in the respiratory mucosa, and on the other hand, to characterize the beneficial effect in terms of its efficacy in protecting against different pathogens, as well as its potential to modulate the immune system of different hosts such as children, the elderly, and immunocompromised patients [6]. This work aimed to evaluate whether orally administered *L. rhamnosus* CRL1505 conferred protection against respiratory superinfection and the duration of such protection. For this purpose, we used models of Toll-like receptor 3 (TLR3)-mediated lung inflammation or RSV infection followed by the secondary infection with *S. pneumoniae* at different time points in young mice. The impact of the CRL1505 strain on the susceptibility to secondary *Streptococcus pneumoniae* infection and the respiratory innate immune response, particularly focused on AMphs, was evaluated.

## 2. Materials and Methods

### 2.1. Lactobacilli Strains

*L. rhamnosus* CRL1505, *L. rhamnosus* CRL498 and *L. plantarum* CRL1506 belong to the CERELA-CONICET culture collection (Tucuman, Argentina). *L. rhamnosus* IBL027 belongs to the Infection Biology Laboratory of INSIBIO-CONICET (Tucuman, Argentina), and *L. plantarum* MPL16 belongs to the Laboratory of Animal Food Function, Tohoku University (Sendai, Japan). Lactobacilli were grown in Man–Rogosa–Sharpe (MRS) broth medium (Oxoid, Hampshire, UK) for 15 h at 37 °C (final log phase). Centrifugation at 3000× *g* for 10 min was used to harvest microorganisms, which then were washed three times with sterile 0.01 mol/L phosphate-buffered saline (PBS, pH 7.2) and suspended in sterile non-fat milk (10%) for administration to the animals.

### 2.2. Animals and Treatments

Female three-week-old BALB/c mice were sourced from the animal facilities of CERELA-CONICET (San Miguel de Tucumán, Argentina). The mice were housed in plastic cages, and each experimental parameter was evaluated using groups of 5–6 mice per time point. Lactobacilli strains were delivered for five consecutive days at a dosage of 10^8^ cells per mouse per day via drinking water. This dose was previously selected as the optimal dose to induce immunomodulation [6,8]. Both the treated and the control mice, which received non-fat milk, were given a standard balanced diet ad libitum. Animals were stimulated with poly(I:C) or infected with RSV, and then challenged with pneumococci at days 5, 10, 15 or 20 after the last poly(I:C) administration or RSV infection (Supplementary Figure S1), as described below. The study adhered to the guidelines set by the CERELA-CONICET Institutional Animal Care and Use Committee and followed the Guide for the Care and Use of Laboratory Animals. The experimental protocols (CRL-CICUAL-IBT-2024/3A and CRL-CICUAL-IBT-2024/7A) received prior approval from the committee. Every effort was made to minimize animal usage and distress, with no observed signs of pain or discomfort before reaching experimental endpoints. No mortality was observed during the study.

### 2.3. Poly(I:C) Administration and Respiratory Infections

Poly(I:C) was administered to the mice one day after the final day of oral lactobacilli treatments. A dose of 250 μg poly(I:C) in 100 μL of PBS was delivered intranasally, which corresponds to 10 mg/kg body weight. This was repeated three times with 24 h intervals between each dose.

Vero cells were used to propagate human RSV strain A2, following established protocols. The cells were cultured in Dulbecco’s Modified Eagle’s Medium (DMEM) and infected with RSV for 3 h at 37 °C and 5% CO_2_. Afterward, DMEM with 10% fetal bovine serum (FBS), 0.1% penicillin–streptomycin, and 0.001% ciprofloxacin was added to allow for syncytium formation. The virus was purified through a sucrose density gradient and stored at −80 °C. For challenge experiments, animals were infected intranasally with 10^6^ plaque-forming units (PFU) of the virus one day after completing lactobacilli treatment. Lung RSV titers were measured at day 2 post-infection using an immunoplaque assay. Primary antibodies targeting RSV-F and RSV-G glycoproteins and a secondary horseradish peroxidase-linked anti-mouse IgG antibody were used to develop individual plaques. Results are shown as log_10_ PFU/g of lung tissue [12].

*Streptococcus pneumoniae* serotype 6B was provided by the National Institute of Infectious Diseases of the Argentinean Government (ANLIS-Malbran, Buenos Aires, Argentina), which characterized the strain by serotyping and molecular biology. The bacterium was cultured on blood agar for 18 h at 37 °C in 5% CO_2_. Pneumococcal colonies were then suspended in Todd Hewitt broth. Following overnight incubation at 37 °C and 5% CO_2_, the bacteria were harvested, washed, and resuspended in PBS at a cell density of 4 × 10^7^ CFU/mL. Mice were challenged with pneumococci at various time points (5, 10, 15, or 20 days) post-poly(I:C) or RSV infection. For infection, 10^3^ pneumococcal cells were administered to each mouse according to our previous work [12]. Mice were infected intranasally with 25 µL of pathogen suspension. Two days after infection, mice were euthanized, and their lungs were homogenized and plated on blood agar to quantify *S. pneumoniae* growth. Results were recorded as log CFU per gram of lung tissue or CFU per mL of blood [12].

### 2.4. Lung Injury Markers

Broncho-alveolar lavage (BAL) samples were collected following previously established protocols [12]. In brief, the trachea was cannulated using a catheter, followed by two consecutive lavages with sterile PBS. The BAL fluid was centrifuged at 900× *g* for 10 min, and the supernatant was stored at −80 °C for later analysis.

To assess lung injury, total protein and albumin concentrations and lactate dehydrogenase (LDH) activity were measured in BAL samples. Proteins were quantified by the bicinchoninic protein assay kit (Pierce Biotechnology Inc., Rockford, IL, USA) as described previously [12]. Albumin levels were quantified using a colorimetric assay based on bromocresol green binding, employing an Albumin Diagnostic Kit (Wiener Lab, Buenos Aires, Argentina). LDH activity was measured as the production of NADH, following the procedures and reagents provided by Wiener Lab. Briefly, 20 µL of BAL samples were incubated at 30 °C with 1 mL of reactive A: Tris HCl (80 mM, pH 7.2), pyruvate (1.6 mmol/L), NADH (0.2 mmol/L), and NaCl (200 mmol/L). The initial absorbance (340 nm) and the absorbance at 1, 2, and 3 min were read. The average absorbance difference/min (∆A/min) was determined, and the results are shown in units per liter of BAL fluid, following the table correction provided by the kit.

Neutrophils counts in BAL samples were also assessed as a measure of inflammation. The total leukocyte counts in BAL samples were measured using a hemocytometer, while differential counts were performed with May Grünwald–Giemsa stain [12]. Total leukocyte counts and neutrophil percentages in smears were used to calculate the absolute number of BAL neutrophils.

### 2.5. AMphs Primary Cultures

Primary cultures of AMphs from mice were prepared following previously described methods [12]. Macrophages were isolated through bronchoalveolar lavages using 1 mL of PBS with 5 mM EDTA. The collected cells were washed twice with PBS and then resuspended in RPMI 1640 medium supplemented with 10% FBS, 1 mM L-glutamine, and 100 U/mL penicillin–streptomycin. In order to allow for adherence, cells were cultured (10^5^ cells per well) in 24-well plates and incubated (37 °C, 5% CO_2_) for 2 h. Non-adherent cells were removed by washing, and the remaining macrophages were cultured in RPMI 1640 medium with 10% FBS, 1 mM L-glutamine, and 100 U/mL penicillin–streptomycin for 24 h (37 °C, 5% CO_2_) prior to stimulation. AMphs were then stimulated with *S. pneumoniae* at an MOI = 5. Supernatants before (baseline) and 24 h after stimulation were collected for cytokine determinations.

### 2.6. Cytokine Concentrations

Cytokine levels, including IFN-β, IFN-γ, IL-6, IL-10, IL-12, and IL-27 (Mouse Quantikine ELISA Kits), were determined in BAL samples and culture supernatants using enzyme-linked immunosorbent assay (ELISA) kits, following the manufacturer’s protocols (R&D Systems, MN, USA). Additionally, CCL2 (Mouse MCP1 ELISA Kit) and TNF-α (Mouse TNF alpha ELISA Kit) levels were measured using ELISA kits from Abcam (Cambridge, UK), adhering to the manufacturer’s instructions. The sensitivity values for IFN-β, IFN-γ, IL-6, IL-10, IL-12, IL-27, CCL2, and TNF-α were 15.5, 2, 1.8, 5.2, 1.5, 4.7, 0.487, and 9.1 pg/mL, respectively.

### 2.7. Flow Cytometry Analysis

Cells from BAL samples were isolated as described previously [12]. Cells were counted with the Trypan Blue method and suspended at a cell density of 5 × 10⁶ cells/mL. To prevent nonspecific binding, BAL cells were treated with anti-mouse CD32/CD16 monoclonal antibody (Fc block, 15 min, 4 °C). Subsequently, cells were treated with a mix of fluorochrome-conjugated antibodies (30 min, 4 °C), followed by washes with FACS buffer. The antibodies used for staining included CD45 (FITC mark), CD11c (APC mark), and SiglecF (PE mark) from BD Bioscience (San Jose, CA, USA) and MHC-II (PerCP mark) from Thermo Fisher Scientific (Waltham, MA, USA). The stained cells were processed with a BD FACSCalibur™ flow cytometer (BD Biosciences, Franklin Lakes, NJ, USA), and the data were analyzed using FlowJo software Version V10 (TreeStar, Ashland, OR, USA). To determine the total number of cells in each population, the percentages of marker-positive and negative cell subsets were multiplied by the total cell number from each BAL sample.

### 2.8. Statistical Analysis

All experiments were conducted in triplicate. The results are presented as means with standard deviations. Data distribution was checked for normality prior to statistical analysis. A two-way ANOVA was applied to assess the differences between groups, followed by Tukey’s test for pairwise comparisons. *p* < 0.05 was selected for statistical significance.

## 3. Results

### 3.1. Orally Administered L. rhamnosus CRL1505 Enhances Resistance Against Respiratory Superinfection

**Figure 1:**
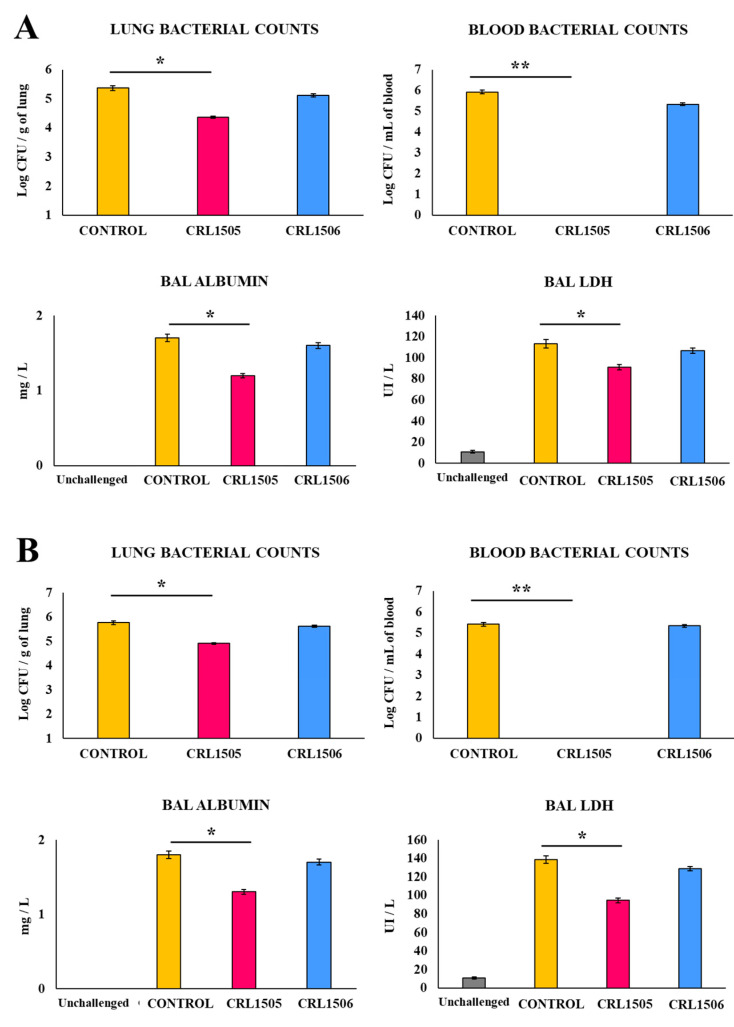
Effect of *Lacticaseibacillus rhamnosus* CRL1505 and *Lactiplantibacillus plantarum* CRL1506 on respiratory superinfection. Infant mice were fed *L. rhamnosus* CRL1505 or *L. plantarum* CRL1506 for 5 days and stimulated with poly(I:C) on days 7, 8, and 9 (**A**) or challenged with respiratory syncytial virus (RSV) on day 7 (**B**) via the nasal route. Five days later, mice were nasally infected with *Streptococcus pneumoniae*. The pneumococcal cell counts in lung and blood, the concentration of BAL albumin, and the activity of BAL LDH were determined 2 days after *S. pneumoniae* infection. The results are shown as mean ± SD. Significant differences are shown compared to the control group at *p* < 0.05 (*) or *p* < 0.01 (**).

The effect of the oral treatment of young mice with the immunobiotic strain *L. rhamnosus* CRL1505 on their resistance to secondary pneumococcal pneumonia was first evaluated. Secondary bacterial infection was induced after the lung TLR3-mediated inflammation of RSV infection. In both models, 100% of mice in the control group were dead at day 8 after the challenge with pneumococci, while 60% and 50% of the animals in the CRL1505-treated group were alive at day 8 after the poly(I:C)–pneumococci and RSV–pneumococci challenges, respectively. We also assessed the pneumococcal colonization and dissemination as well as lung damage two days after the infection with the respiratory pathogen in the poly(I:C) (Figure 1A) and the RSV (Figure 1B) models. In these experiments, groups of mice fed the immunobiotic strain *L. plantarum* CRL1506, which, when orally administered, is not able to increase the resistance of mice to primary infections with *S. pneumoniae*, RSV, or IFV [6,9], were used for comparisons. As we described previously [12], in control mice which received poly(I:C) (Figure 1A) or RSV (Figure 1B) and were then infected with *S. pneumoniae*, the bacterial pathogen was observed in the blood and lungs.

**Figure 2:**
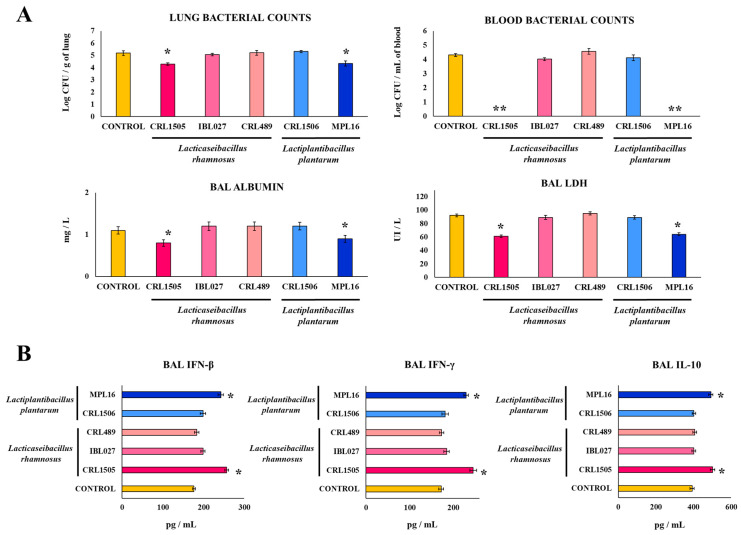
Effect of lactobacilli on respiratory superinfection. Infant mice were fed *L. rhamnosus* CRL1505, IBL027, CRL489, *L. plantarum* CRL1506, or MPL16 for 5 days and stimulated with poly(I:C) on days 7, 8, and 9 via the nasal route. Five days later, mice were nasally infected with *Streptococcus pneumoniae*. The pneumococcal cell counts in lung and blood, the concentration of BAL albumin, the activity of BAL LDH (**A**), and the concentrations of BAL IFN-β, IFN-γ, and IL-10 (**B**) were determined 2 days after *S. pneumoniae* infection. The results are shown as mean ± SD. Significant differences are shown compared to the control group at *p* < 0.05 (*) or *p* < 0.01 (**).

In addition, increases in the biochemical markers of lung injury BAL protein, albumin, and LDH were observed in controls (Figure 1 and Figure S2). Mice treated with the CRL1505 strain before poly(I:C) stimulation and the infection with pneumococci had significantly lower lung bacterial cell counts, BAL protein, and albumin concentrations and BAL LDH activity (Figure 1A and Figure S2A). Furthermore, pneumococci were not found in blood samples of animals treated with *L. rhamnosus* CRL1505. When respiratory superinfection was performed by replacing the poly(I:C) stimulus with a challenge with RSV, similar results were observed. The CRL1505 strain significantly reduced RSV titers in the lungs (Figure S3), diminished lung pneumococcal cell counts, reduced lung injury biomarkers, and avoided the dissemination of *S. pneumoniae* into the blood (Figure 1B and Figure S2B). As expected, *L. plantarum* CRL1506-treated young mice showed resistance to RSV, with lung and blood pneumococcal counts as well as values of BAL albumin concentrations and BAL LDH activity that were not significantly different from the controls in both the poly(I:C)–pneumococci (Figure 1A and Figure S2A) and RSV–pneumococci (Figure 1B, Figure S2B, Figure S3) respiratory superinfection models. The numbers of BAL neutrophils were also assessed as inflammation markers in both models of respiratory superinfection. BAL neutrophil counts were augmented in all the experimental groups; however, the CRL1505-treated mice had significantly lower neutrophil counts than controls (Figure 2).

We also determined whether other strains of the same species, with or without immunomodulatory properties, affected the susceptibility of young mice to the respiratory superinfection. Then, distinct groups of animals were fed *L. rhamnosus* IBL027, *L. rhamnosus* CRL489, and *L. plantarum* MPL16,; stimulated with poly(I:C); and infected with *S. pneumoniae*. The resistance of these mice to secondary pneumococcal infection was compared with controls and with animals receiving *L. rhamnosus* CRL1505 and *L. plantarum* CRL1506 (Figure 2). The probiotic strain MPL16 [6] could reduce the lung pneumococcal cell counts, diminish BAL albumin LDH levels, and avoid the dissemination of *S. pneumoniae* into the blood in a way similar to that observed for *L. rhamnosus* CRL1505 (Figure 2A). In contrast, the immunomodulatory strain IBL027 [6] was not able to modify the resistance to the secondary pneumococcal pneumonia. As expected, mice treated with the non-immunomodulatory *L. rhamnosus* CRL489 had pneumococcal counts and values of BAL albumin and LDH that were not significantly different from controls (Figure 2A). We previously demonstrated that the increased protection against respiratory superinfection induced by nasally administered immunobiotics is related to a differential modulation of IFN-β, IFN-γ, and IL-10 levels in the respiratory mucosa [12]. Thus, we determined the concentrations of these three cytokines in BAL samples from control and lactobacilli-treated groups on day two post-infection (Figure 2B). The levels of BAL IFN-β, IFN-γ, and IL-10 in mice receiving CRL1505 and MPL16 strains were significantly higher than the controls and the groups of mice fed *L. rhamnosus* IBL027, *L. rhamnosus* CRL489, or *L. plantarum* CRL1506 (Figure 2B).

### 3.2. AMphs’ Role in the Improved Resistance Against Respiratory Superinfection Induced by Orally Administered Immunobiotics

**Figure 3:**
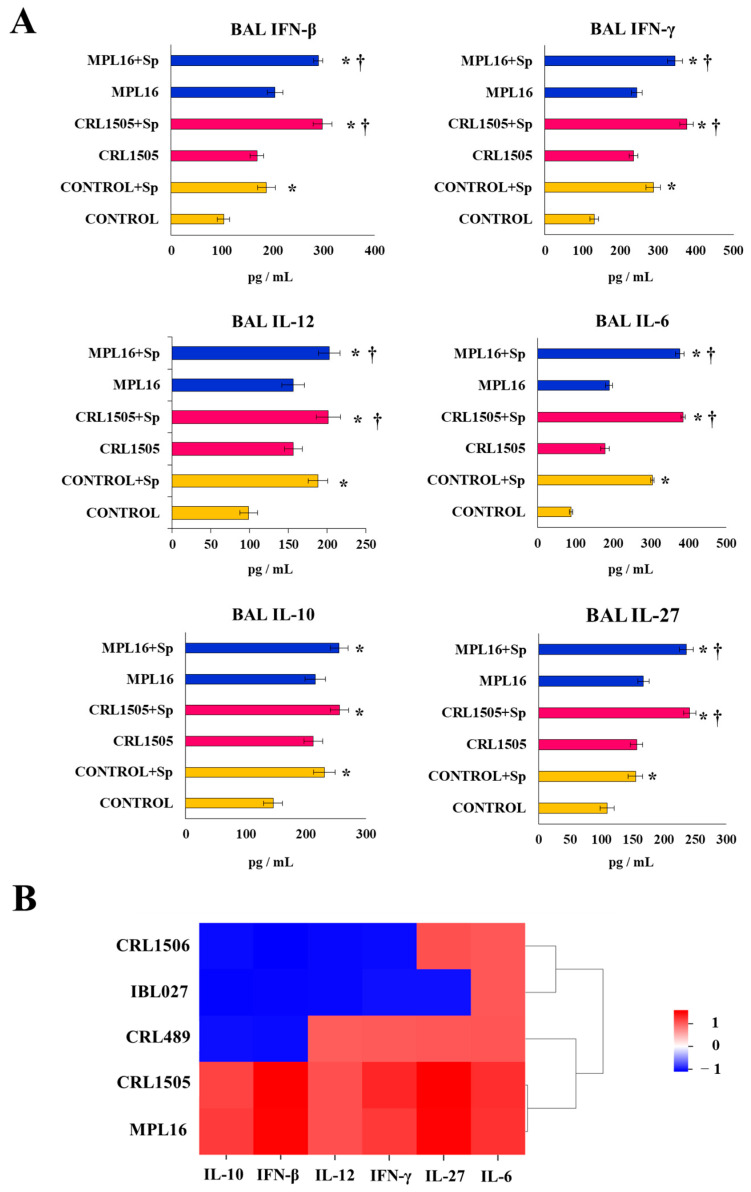
Effect of lactobacilli on AMphs cytokine production. Infant mice were fed *L. rhamnosus* CRL1505, IBL027, CRL489, *L. plantarum* CRL1506, or MPL16 for 5 days and stimulated with poly(I:C) on days 7, 8, and 9 via the nasal route. Five days later, AMphs were isolated from BAL samples, cultured, and in vitro challenged with *Streptococcus pneumoniae*. The concentrations of IFN-β, IFN-γ, IL-6, IL-10, IL-12, and IL-27 were evaluated on AMph supernatants after 24 h. (**A**) Cytokine production of AMphs from *L. rhamnosus* CRL1505 and *L. plantarum* MPL16. The results are shown as mean ± SD. Significant differences were shown compared to the respective basal levels without pneumococcal challenge at *p* < 0.05 (†). Significant differences were shown compared to the control group at *p* < 0.05 (*). (**B**) Heatmap shows the variations in the concentration of cytokines of all experimental groups in relation to the control.

To investigate the specific participation of AMphs in the immunomodulatory effects of *L. rhamnosus* CRL1505 and *L. plantarum* MPL16, we prepared primary cultures of these cells obtained from the control and lactobacilli-treated young mice. These primary cultures of AMphs were stimulated with *S. pneumoniae* (Figure 3A). The interferons IFN-β and IFN-γ, as well as the cytokines IL-6, IL-12, IL-10, and IL-27, were detected in primary cultures of mouse AMphs as described previously [12]. In addition, it was observed that the basal levels of all these immune factors were significantly higher in AMphs cultures from CRL1505- and MPL16-treated animals than in the control mice (Figure 3A). The infection with *S. pneumoniae* augmented the concentrations of both IFNs (IFN-β and IFN-γ), the inflammatory cytokines IL-6 and IL-12, as well as the immunoregulatory cytokines IL-27 and IL-10 in AMphs cultures. Notably, macrophages obtained from young mice receiving *L. rhamnosus* CRL1505 and *L. plantarum* MPL16 produced higher levels of IFNs, IL-6, IL-12, and IL-27 than cells from control animals (Figure 3A), while IL-10 was not different from the control macrophages. When the production of cytokines in AMphs primary cultures from mice fed *L. rhamnosus* IBL027, *L. rhamnosus* CRL489, or *L. plantarum* CRL1506 were compared to controls, no differences were found for any of the evaluated cytokines (Figure 3B).

**Figure 4:**
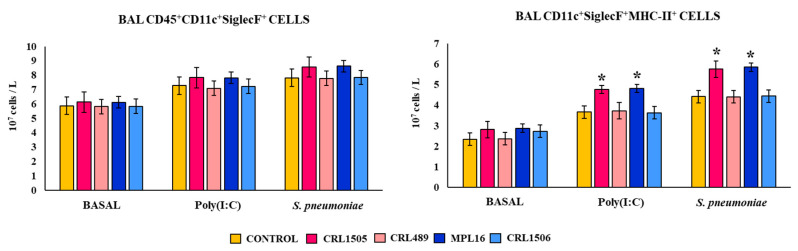
Effect of lactobacilli on AMphs MHC-II expression. Infant mice were fed *L. rhamnosus* CRL1505, IBL027, CRL489, *L. plantarum* CRL1506, or MPL16 for 5 days and stimulated with poly(I:C) on days 7, 8, and 9 via the nasal route. Five days later, mice were nasally infected with *Streptococcus pneumoniae*. The numbers of CD45^+^CD11c^+^SiglecF^+^ and CD11c^+^SiglecF^+^MHC-II^+^ cells in BAL were determined on the last day of lactobacilli treatment (basal) and 2 days after poly(I:C) stimulation and *S. pneumoniae* infection. The results are shown as mean ± SD. Significant differences are shown compared to the control group at *p* < 0.05 (*).

We also assessed the effect of lactobacilli on the expression of MHC-II on AMphs. The total BAL CD45^+^CD11c^+^SiglecF^+^ AMphs population and BAL CD11c^+^SiglecF^+^MHC-II^high^ macrophages were studied by flow cytometry one day after the oral treatments with *L. rhamnosus* CRL1505 and *L. plantarum* MPL16, two days after the last poly(I:C) stimulation, and two days after the pneumococcal infection (Figure 4 and Figure S4).

The total numbers of BAL CD45^+^CD11c^+^SiglecF^+^ AMphs were not changed by the feeding with *L. rhamnosus* CRL1505 or *L. plantarum* MPL16. The AMphs numbers were enhanced upon the stimulation with poly(I:C) and *S. pneumoniae* infection (Figure 4), but no differences were detected between the CRL150- and MPL16-treated mice and controls within the same period. As we reported previously [12], in basal conditions, less than 25% of AMphs are MHC-II^high^ cells, with most of them being MHC-II^low^ cells. When the CD11c^+^SiglecF^+^MHC-II^high^ AMphs population was evaluated, it was detected that the numbers of these cells increased after the stimulation with poly(I:C) and *S. pneumoniae* infection in all the experimental groups (Figure 4 and Figure S4). However, more CD11c^+^SiglecF^+^MHC-II^high^ cells were observed in mice receiving CRL1505 or MPL16 strains when compared to the controls.

### 3.3. Long-Term Protection Induced by Orally Administered Immunobiotics Against Respiratory Superinfection

**Figure 5:**
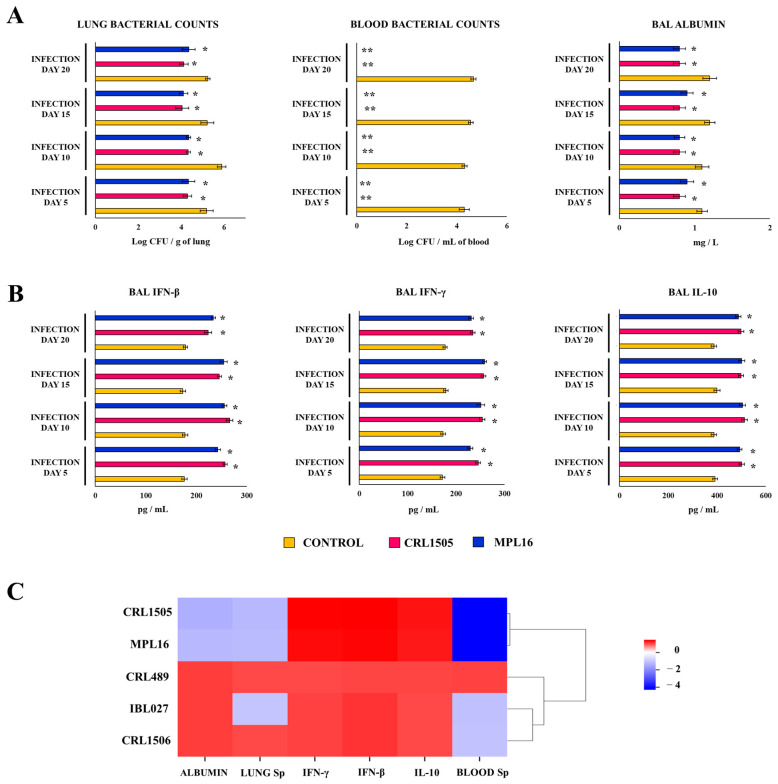
Effect of lactobacilli on respiratory superinfection. Infant mice were fed *L. rhamnosus* CRL1505, IBL027, CRL489, *L. plantarum* CRL1506, or MPL16 for 5 days and stimulated with poly(I:C) on days 7, 8, and 9 via the nasal route. For the evaluation of long-term protection, 5, 10, 15, or 20 days after the last administration of poly(I:C), mice were nasally infected with *Streptococcus pneumoniae*. The pneumococcal cell counts in lung and blood, the concentration of BAL albumin, the activity of BAL LDH (**A**), and the concentrations of BAL IFN-β, IFN-γ and IL-10 (**B**) were determined 2 days after *S. pneumoniae* infection. The results are shown as mean ± SD. Significant differences are shown compared to the control group at *p* < 0.05 (*) or *p* < 0.01 (**). (**C**) Heatmap shows the variations in the parameters evaluated for all experimental groups in relation to the controls.

Next, we evaluated the duration of the beneficial effect achieved by immunomodulatory lactobacilli in the context of respiratory superinfection (Figure 5). Then, young mice were orally treated with *L. rhamnosus* CRL1505 or *L. plantarum* MPL16, stimulated with poly(I:C), and infected with *S. pneumoniae* 10, 15, or 20 days after the last administration of the TLR3 agonist. The resistance to the infection, the lung damage, and the levels of respiratory IFNs and IL-10 were determined and compared with those obtained from experiments in which pneumococcal infection was produced 5 days after poly(I:C) stimulation (Figure 5). The lung and blood pneumococcal counts, as well as the values of BAL albumin concentrations in CRL1505- and MPL16-treated mice, were significantly lower than controls at all the time points evaluated and with no differences between them (Figure 5A). Similarly, the concentrations of BAL IFNs and IL-10 were higher in mice fed *L. rhamnosus* CRL1505 or *L. plantarum* MPL16 than in controls in all the time points evaluated, with no differences between them (Figure 5B). When the resistance to pneumococcal infection; the lung injury; and the levels of BAL IFN-β, IFN-γ, and IL-10 from young animals receiving *L. rhamnosus* IBL027, *L. rhamnosus* CRL489, or *L. plantarum* CRL1506 and challenged with pneumococci 20 days after poly(I:C) administration were compared to controls, no differences were found for any of the evaluated parameters (Figure 5C).

In light of these results, we wonder whether the treatment with the CRL1505 and MPL16 strains could provide long-term protection in the context of a primary pneumococcal infection. For this purpose, distinct groups of mice were fed *L. rhamnosus* CRL1505 or *L. plantarum* MPL16 and then infected with *S. pneumoniae* 1, 5, 7, or 10 days after the last administration of lactobacilli (Figure S5). Lung and blood pneumococcal counts and the levels of BAL albumin were determined (Figure S6). Both CRL1505 and MPL16 were capable of significantly reducing *S. pneumoniae* lung colonization and the concentrations of BAL albumin as well as avoiding the pathogen’s dissemination the into the blood when infection was produced 1, 5, or 7 days after the treatment with lactobacilli. On the contrary, the values of all the parameters evaluated were not different between CRL1505 and MPL16 treatment and controls when the pneumococcal challenge was produced 10 days after the last administration of lactobacilli (Figure S6).

### 3.4. Orally Administered Immunobiotics Improve the Kinetics of Innate Immunity Against Secondary Pneumococcal Pneumonia

**Figure 6:**
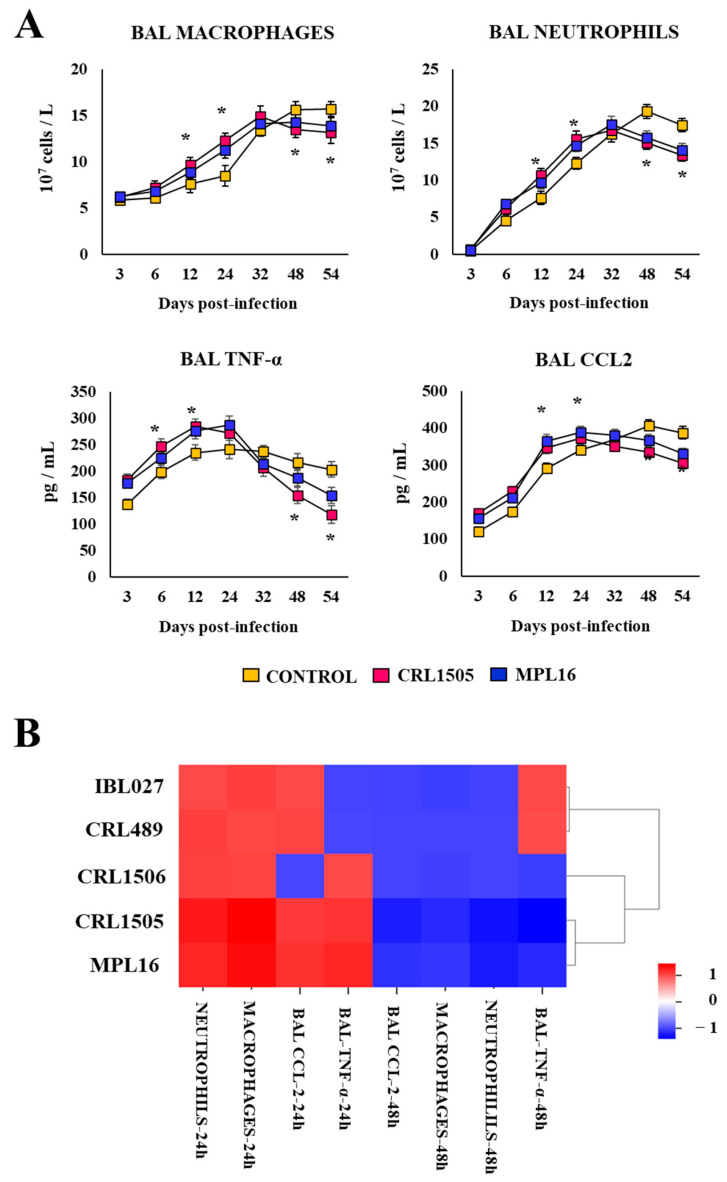
Effect of lactobacilli on respiratory superinfection. Infant mice were fed *L. rhamnosus* CRL1505, IBL027, CRL489, *L. plantarum* CRL1506, or MPL16 for 5 days and stimulated with poly(I:C) on days 7, 8, and 9 via the nasal route. Twenty days after the last administration of poly(I:C), mice were nasally infected with *Streptococcus pneumoniae*. (**A**) The number of macrophages and neutrophils and the concentrations of TNF-α and CCL2 in BAL samples were determined 3, 6, 12, 24, 32, 48, and 54 h after *S. pneumoniae* infection. The results are shown as mean ± SD. Significant differences are shown compared to the control group at *p* < 0.05 (*) (**B**) Heatmap shows the variations in the parameters evaluated at hours 24 and 48 of all experimental groups in relation to the control.

Finally, we aimed to evaluate the influence of orally administered immunobiotics on the kinetics of the innate immune response to secondary pneumonia in greater depth. Then, infant mice were fed *L. rhamnosus* CRL1505 or *L. plantarum* MPL16, stimulated with poly(I:C), and then challenged with *S. pneumoniae* 20 days after the last administration of the TLR3 agonist. BAL macrophages and neutrophils as well as respiratory TNF-α and CXCL2 levels were determined at several points after the *S. pneumoniae* infection (Figure 6A).

Infant mice treated with CRL1505 or MPL16 strains had an earlier peak of BAL neutrophil and macrophage counts and TNF-α and CXCL2 concentrations. Immune cells showed the highest values between 24 and 32 h in lactobacilli-treated animals, while the inflammatory cytokines showed the highest values between 12 and 24 h. Both immune cells and cytokines showed a decreasing trend until hour 54 (Figure 6A). Notably, a delayed increase in inflammatory cells and cytokines was observed in the control mice when compared to lactobacilli-fed animals. Furthermore, the numbers of BAL immune cells and the levels of TNF-α and CXCL2 were significantly lower in *L. rhamnosus* CRL1505- and *L. plantarum* MPL16-treated mice between hours 48 and 54 than in controls (Figure 6A).

When BAL macrophages, neutrophils, TNF-α, and CXCL2, determined at hours 24 and 48 post-pneumococcal challenge from mice treated with *L. rhamnosus* IBL027, *L. rhamnosus* CRL489, or *L. plantarum* CRL1506, were contrasted to controls, no differences were found for any of the evaluated parameters (Figure 6B).

## 4. Discussion

The impact of orally administered *L. rhamnosus* CRL1505 on the resistance and the immune response against primary infections with *S. pneumoniae*, RSV, and IFV has been widely characterized [6,9,12]. In addition, we showed previously that the purified peptidoglycan of the CRL1505 strain, administered nasally, enhanced the resistance of mice to secondary pneumococcal infection through trained AMphs development [12]. However, the effect of orally administered *L. rhamnosus* CRL1505 in the context of respiratory superinfection has not been evaluated before. In this work, we showed that the CRL1505 strain is capable of increasing the resistance to the secondary pneumococcal pneumonia produced after TLR3 activation or RSV infection through modulation of the gut–lung axis. We observed that feeding *L. rhamnosus* CRL1505 can confer long-term protection against secondary *S. pneumoniae* infection and that this phenomenon would be mediated by the generation of trained AMphs. Furthermore, we found that the immunomodulatory strain *L. plantarum* MPL16 [6] has a similar effect to the CRL1505 in the context of respiratory superinfections.

Previously, we described that pneumococcal infection is more severe after the TLR3 activation or RVS infection [12]. For the induction of pneumonia in BALB/c mice after a primary challenge, 10^5^–10^6^ *S. pneumoniae* cells per mouse are necessary, while only 10^3^ pneumococcal cells are enough to produce lung infection in mice previously treated with poly(I:C) or challenged with RSV. In line with our findings, it was reported that RSV infection in BALB/c mice significantly altered *S. pneumoniae* clearance [13]. More severe secondary pneumococcal infection has been also described in mouse models of IFV-*S. pneumoniae* superinfection. When mice superinfected with IFV followed by a challenge with pneumococci 5 days later were compared with mice infected with IFV or *S. pneumoniae* only, a higher respiratory disease severity was shown for the superinfection than for the individual challenges [2]. Superinfected animals had significantly higher weight loss, lung pathogens loads, and inflammatory factors in comparison with animals infected only with IFV or *S. pneumoniae*. Notably, the treatment of infant mice with *L. rhamnosus* CRL1505 or *L. plantarum* MPL16 significantly reduced the severity of secondary pneumococcal infection, as shown by the lower levels of the biochemical markers of lung injury and inflammation, the reduced pathogen counts, and the avoidance of pneumococcal dissemination into blood. These effects were related to a distinct regulation of the respiratory immune response.

Consistent with our previous findings in poly(I:C) and RSV infection models [6], increased levels of respiratory IFN-β and IFN-γ were detected in mice receiving CRL1505 or MPL16 strains after the secondary *S. pneumoniae* challenge. These cytokines are known to participate in the protection against the pathogen. Studies have shown that macrophages produce type I IFNs in response to *S. pneumoniae*, and studies in animal models have confirmed that AMphs are the main source of these IFNs upon infection with the pneumococci [14]. Type II pneumocytes stimulated by type I IFNs synthetized by AMphs have increased protection against pneumococcal infection and cell death [14]. Additionally, IFN-β enhances alveolar barrier function and provides protection against invasive pneumococcal disease [15,16,17]. Furthermore, IFN-γ also has a significant role in the protection against *S. pneumoniae*. Higher levels of IFN-γ stimulate pulmonary macrophages [18] and increase the expression of IFN-γ-related genes in the lung tissue [19], mechanisms that are critical for defending against pneumococcal infections. Studies that have compared RSV infection in young and adult mice have revealed that differences in IFN-γ synthesis are related to different levels of susceptibility to the respiratory disease [20,21]. Adult mice efficiently recruited CD8^+^ and CD4^+^ T cells expressing IFN-γ to the lungs, leading to AMphs activation and effective clearance of RSV. In contrast, young mice could only recruit CD8^+^IFN-γ^+^ T cells to the respiratory tract, and the lack of efficient recruitment of CD4^+^ T cells expressing IFN-γ and AMphs activation were associated with augmented lung RSV titers.

It was also observed that CRL1505- and MPL16-treated mice differentially regulated the pro- and anti-inflammatory cytokine balance in the respiratory tract at the time of the secondary pneumococcal pneumonia. It has been demonstrated that TNF-α is essential for clearing the virus during the generation of the innate immune response to RSV. However, sustained synthesis of TNF-α can augment disease severity and lead to tissue damage in the later stages of infection [22]. In addition, it was shown that AMph depletion before *S. pneumoniae* infection leads to increased lung inflammation and augmented mortality because of the failure in the production of anti-inflammatory cytokines like IL-10 [23]. The different TNF-α production kinetics in the lungs and the increased production of IL-6 and IL-27 by AMphs in young mice receiving the CRL1505 or MPL16 strains suggests that an appropriate balance of pro-inflammatory and regulatory factors participates in the improved clearance of pneumococci and the protection against lung inflammatory-mediated injury at the time of *S. pneumoniae* secondary infection, as previously observed in the context of primary pneumococcal and RSV infections [6,11].

It has been reported that, following a primary immunological stimulation, macrophages can develop a nonspecific immune memory that enhances their response to posterior challenge, whether related or not to the first one [24,25]. This trained immunity (also known as innate immune memory) has been associated with increased resistance to pathogens in the respiratory tract [26]. Several findings of our study allow us to speculate that treatments with the strains CRL1505 and MPL16 induced the generation of trained AMphs. It was shown that CD11c^+^SiglecF^+^ AMphs can acquire the trained phenotype from days 5 to 7 after the primary stimulus that was characterized by increased MHC-II expression [26,27]. In our studies, CD45^+^CD11c^+^SiglecF^+^ AMphs from CRL1505 or MPL16 orally treated mice significantly increased their MHC-II expression two days after poly(I:C) and *S. pneumoniae* challenges, corresponding to days 7 and 12 of the experiment. In addition, AMphs from CRL1505- and MPL16-treated infant mice showed improved production of IFNs, TNF-α, IL-6, and CCL2, correlating with a stronger and faster immune response to the secondary *S. pneumoniae* infection. The enhanced ability of macrophages to produce immune mediators in front of a heterologous challenge has been also associated with trained AMph generation [24,25,26]. And most importantly, trained immunity has been associated with long-term protection, a property that differentiates activated macrophages from trained ones. In our hands, the treatment of mice with *L. rhamnosus* CRL1505 or *L. plantarum* MPL16 allowed and improved the response against secondary pneumococcal infection up to 20 days after the viral inflammatory response. Although all these findings indicate the generation of innate immune memory in AMphs, other characteristics also define trained cells, such as metabolic reprogramming and epigenetic changes [28]. Studying whether the treatments with the CRL1505 and MPL16 strains indeed induce these modifications in AMphs is an interesting topic for future research.

Notably, the results suggest that the induction of trained immunity on AMphs by lactobacilli treatments requires the primary inflammatory challenge. The feeding of mice with *L. rhamnosus* CRL1505 or *L. plantarum* MPL16 improved protection against primary *S. pneumoniae* infection for only up to 7 days, distinct from the 20 days of protection induced in the context of secondary pneumococcal pneumonia. It is possible to speculate that the combination of lactobacilli treatments and poly(I:C) stimulation (or RSV infection) is necessary to trigger a trained phenotype in AMphs, and that IFN-γ would have a key role in this phenomenon. It was shown that AMphs acquired the trained phenotype during the inflammatory context of acute IFV infection [27]. These AMphs produced higher levels of IL-6, CCL3, CCL4, and G-CSF and improved the resistance to *S. pneumoniae* infection. AMphs that developed in non-inflammatory settings did not exert an improved response against pneumococci. It was also shown that trained AMphs were programmed by respiratory adenovirus infection under direct contact with lung T CD8^+^ cells and in an environment rich in IFN-γ produced by T CD4^+^ cells [26]. Trained AMphs expressed higher levels of MHC-II, augmented their glycolytic metabolism, produced higher neutrophil chemokines upon re-stimulation and enhanced the resistance of mice to secondary *S. pneumoniae* or *E. coli* nasal challenges. The development of trained AMphs by adenovirus challenge was impaired in IFN-γ^−/−^ and IFN-γR^−/−^ mice [26]. Then, it is possible to speculate that the inflammatory environment induced poly(I:C) (or RSV) would stimulate the activation of lung T CD8^+^ cells, which complemented with the T CD4^+^ IFN-γ-producing cells mobilized from the intestine to the lung under the influence of lactobacilli treatments would create the optimal conditions for the generation of trained macrophages.

## 5. Conclusions

In conclusion, this work provides evidence of the ability of some orally administered lactobacilli strains, like *L. rhamnosus* CRL1505 or *L. plantarum* MPL16, to improve the resistance to primary respiratory viral infections and secondary bacterial pneumonia in infant mice. This work also provides a contribution to our understanding of the mechanisms associated with the modulation of the gut–lung axis by beneficial microbes by demonstrating that specific lactobacilli strains, through the stimulation of the common mucosal immune system, are able to support the generation of trained AMphs that confer long-lasting protection against secondary bacterial challenges produced after a primary inflammatory event in the respiratory tract.

## Data Availability

The data presented in this study are available throughout the article.

## Supplementary Materials

The following supporting information can be downloaded at: https://www.mdpi.com/article/10.3390/biom14121600/s1, Figure S1: Experimental protocol of respiratory superinfection model; Figure S2: Effect of lactobacilli on BAL protein concentration and neutrophil counts after secondary pneumococcal infection induced after poly(I:C) stimulation (A) or RSV infection (B); Figure S3: Effect of lactobacilli on primary RSV infection; Figure S4: Effect of lactobacilli on MHC-II expression in AMphs after secondary pneumococcal infection induced after RSV infection; Figure S5: Experimental protocol of long-term protection studies; Figure S6: Effect of lactobacilli on primary *S. pneumoniae* infection.
