# Supplementary material for: Orally Administered Lactobacilli Strains Modulate Alveolar Macrophages and Improve Protection Against Respiratory Superinfection
Source: Biomolecules. 2024 Dec 14;14(12):1600. doi: 10.3390/biom14121600 (PMC11674826; doi:10.3390/biom14121600)
Supplement: Supplementary file 1 [file biomolecules-14-01600-s001.zip › biomolecules-3278043-supplementary.pdf]

biomolecules

MDPI

Supplementary Materials

Orally Administered Lactobacilli Strains Modulate Alveolar Macrophages and Improve Protection Against Respiratory Superinfection

Leonardo Albarracin [1,†], Stefania Dentice Maidana [1,†], Kohtaro Fukuyama [2], Mariano Elean [1], Julio Nicolás Argañaraz Aybar [3], Yoshihito Suda [4], Keita Nishiyama [2,5], Haruki Kitazawa [2,5,*] and Julio Villena [1,*]

[1] Laboratory of Immunobiotechnology, Reference Centre for Lactobacilli (CERELA-CONICET), San Miguel de Tucumán 4000, Argentina; lalbarracin@herrera.unt.edu.ar (L.A.); stefi.dentice@gmail.com (S.D.M.); melean@cerela.org.ar (M.E.)

[2] Food and Feed Immunology Group, Laboratory of Animal Food Function, Graduate School of Agricultural Science, Tohoku University, Sendai 980–8572, Japan; kotaro.fukuyama.p8@dc.tohoku.ac.jp (K.F.); keita.nishiyama.a6@tohoku.ac.jp (K.N.)

[3] Cátedra de Inmunología, Instituto de Microbiología, Facultad de Bioquímica, Química y Farmacia, Universidad Nacional de Tucumán, San Miguel de Tucuman CP4000, Tucumán, Argentina; nic0laz@hotmail.com

[4] Department of Food, Agriculture and Environment, Miyagi University, Sendai 980–8572, Japan; suda@myu.ac.jp

[5] Livestock Immunology Unit, International Education and Research Centre for Food and Agricultural Immunology (CFAI), Graduate School of Agricultural Science, Tohoku University, Sendai 980–8572, Japan;

* Correspondence: haruki.kitazawa.c7@tohoku.ac.jp (H.K.); jcvillena@cerela.org.ar (J.V.)

† These authors contributed equally to this work.

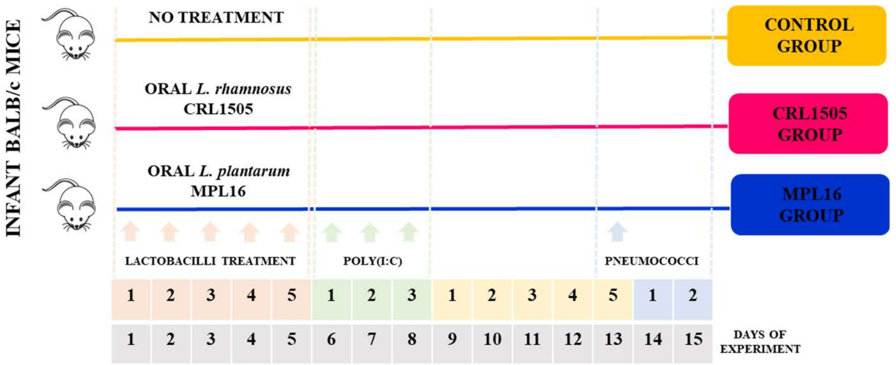

Figure S1. Experimental protocol of respiratory superinfection model.

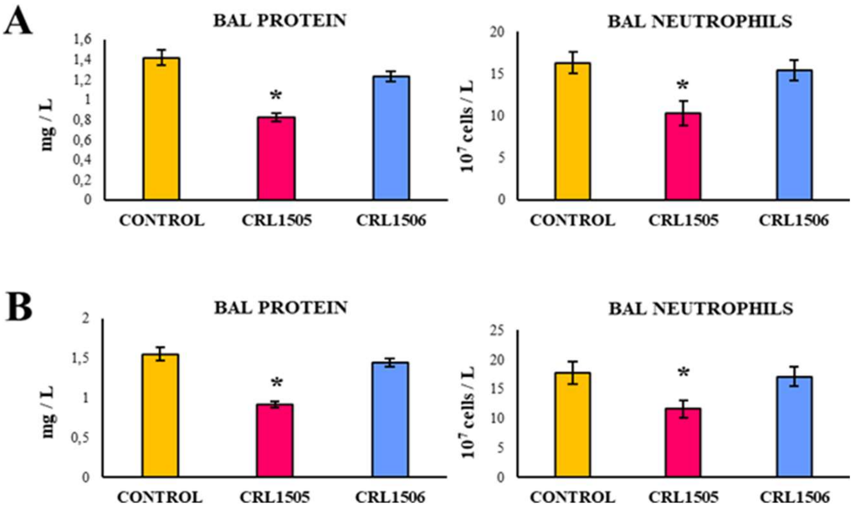

Figure S2. Effect of lactobacilli on BAL protein concentration and neutrophil counts after secondary pneumococcal infection induced after poly(I:C) stimulation (**A**) or RSV infection (**B**).

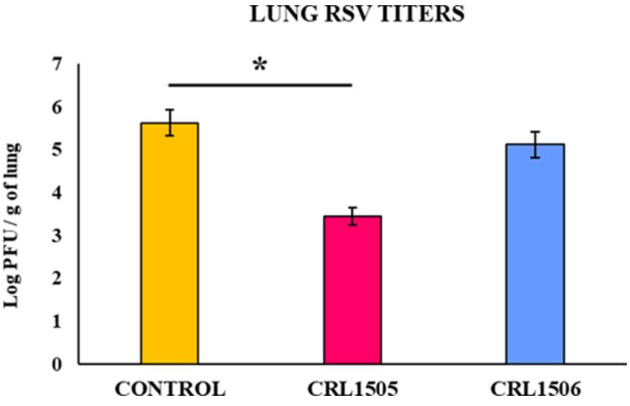

Figure S3. Effect of lactobacilli on primary RSV infection.

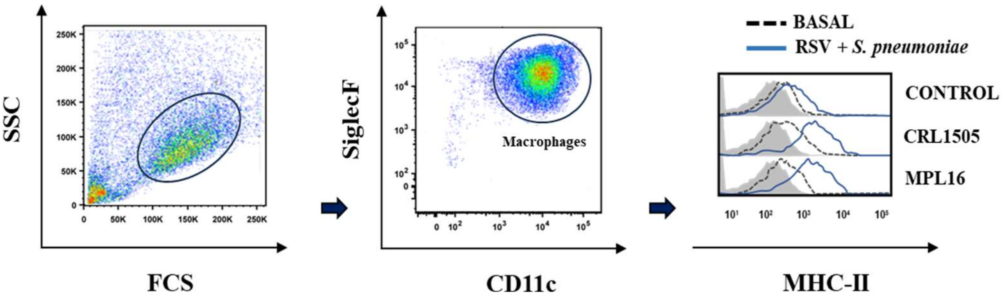

Figure S4. Effect of lactobacilli on MHC-II expression in AMphs after secondary pneumococcal infection induced after RSV infection.

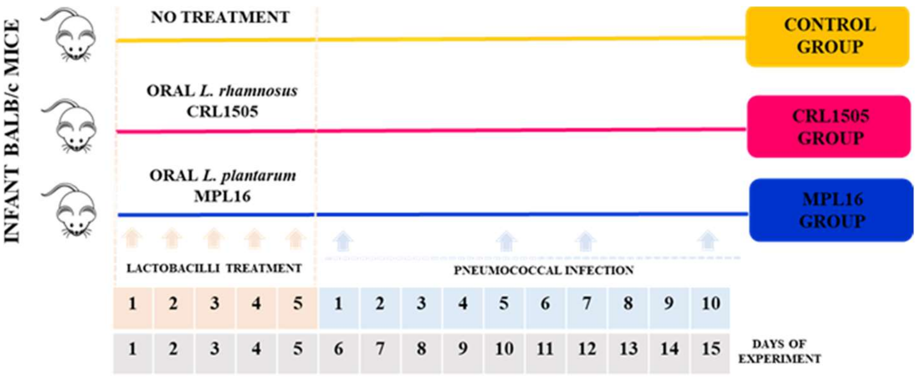

Figure S5. Experimental protocol of long-term protection studies.

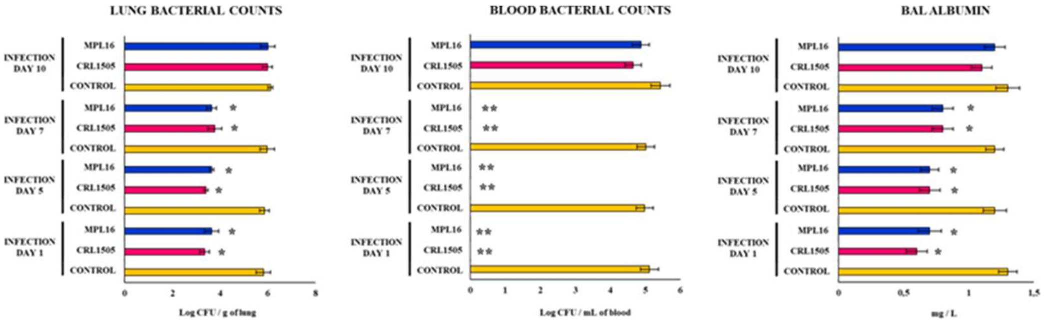

Figure S6. Effect of lactobacilli on primary *S. pneumoniae* infection.
